# Contactless Electric Igniter for Vehicle to Lower Exhaust Emission and Fuel Consumption

**DOI:** 10.1155/2014/795180

**Published:** 2014-02-05

**Authors:** Chih-Lung Shen, Jye-Chau Su

**Affiliations:** ^1^Department of Electronic Engineering, National Kaohsiung First University of Science and Technology, Yanchao, Kaohsiung 824, Taiwan; ^2^Graduate Institute of Engineering Science and Technology, National Kaohsiung First University of Science and Technology, Yanchao, Kaohsiung 824, Taiwan

## Abstract

An electric igniter for engine/hybrid vehicles is presented. The igniter comprises a flyback converter, a voltage-stacked capacitor, a PIC-based controller, a differential voltage detector, and an ignition coil, of which structure is non-contact type. Since the electric igniter adopts a capacitor to accumulate energy for engine ignition instead of traditional contacttype approach, it enhances the igniting performance of a spark plug effectively. As a result, combustion efficiency is promoted, fuel consumption is saved, and exhaust emission is reduced. The igniter not only is good for fuel efficiency but also can reduce HC and CO emission significantly, which therefore is an environmentally friendly product. The control core of the igniter is implemented on a single chip, which lowers discrete component count, reduces system volume, and increases reliability. In addition, the ignition timing can be programmed so that a timing regulator can be removed from the proposed system, simplifying its structure. To verify the feasibility and functionality of the igniter, key waveforms are measured and real-car experiments are performed as well.

## 1. Introduction

Vehicle ignition system can be briefly classified as breaker point ignition system, transistorized ignition system, and capacitor discharge ignition system, of which structures and ignition mechanisms are different from each other [1–5]. However, in common, ignition timing is determined by speed signal generator for all the ignition systems. The speed signal generator mainly consists of a permanent magnet, an inductive coil, and a rotor so as to sense vehicle speed and generate ignition signal. Nevertheless, the speed signal generator cannot accurately produce optimal timing signal and its output voltage is variant. A higher output voltage occurs while in the period of low speed and a lower output voltage while in the period of high speed. It leads to over energy on spark plug in low speed, resulting in energy waste, and also leads to inadequate energy supply in high speed, resulting in knocking.

This paper proposes an engine igniter derived from a flyback-type converter to improve the characteristics of traditional capacitor discharge igniter. The proposed igniter is contactless and powered by battery. With the advantages of microprocessor-based controllers [6–16], the control core of the proposed igniter is designed and implemented on a single chip PIC18F4520. Therefore, the ignition timing is programmable so as to accommodate different vehicle velocities to achieve optimal igniting. Thus, engine can generate the most effective output power and save fuel consumptions significantly. In the igniter, a high-frequency switching flyback converter [17–22] is embedded, which boosts the battery voltage and then accumulates energy on a capacitor. After trigging, the energy stored in the capacitor will be released through a high turns-ratio transformer to ignite spark plug. With the mentioned ignition mechanism, the proposed electric igniter has the following advantages: slowing plug aging, having higher stability for engine operation, having simple structure, being a cost-effective product, improving combustion efficiency, lowering exhaust emission, and saving fuel consumption.

## 2. System Architecture

**Figure 1:**
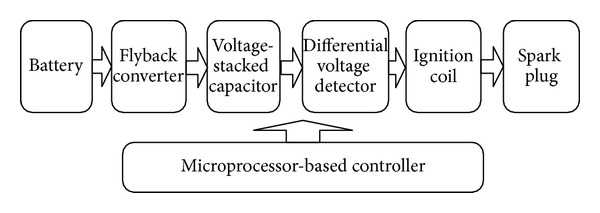
A block diagram to illustrate the structure of the proposed electronic ignition system.

**Figure 2:**
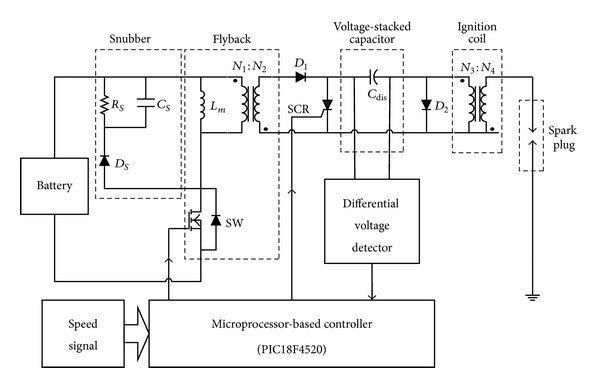
The main circuitry of the proposed igniter.

The block diagram of the proposed capacitor discharge ignition system for engine/hybrid vehicles is shown in Figure 1, which mainly includes a flyback converter, a voltage-stacked capacitor, a microprocessor-based controller, a differential voltage detection circuit, an ignition coil, and a spark plug. The main circuit is shown in Figure 2. The flyback converter is in charge of boosting the battery voltage by means of high-frequency switching and PWM controlling and then continuously stacks up the voltage on the capacitor *C*_dis_ until a voltage level for igniting is reached. The voltage across the voltage-stacked capacitor is detected by the differential voltage detector. After receiving a speed signal, the controller will generate a corresponding trigging signal to turn on the silicon-controlled rectifier (SCR) such that the energy stored in the capacitor *C*_dis_ is discharged to spark plug via the ignition coil. The ignition coil is a high turns-ratio pulse transformer, which upgrades the capacitor voltage to around 15 kV to ignite spark plug.

**Figure 3:**
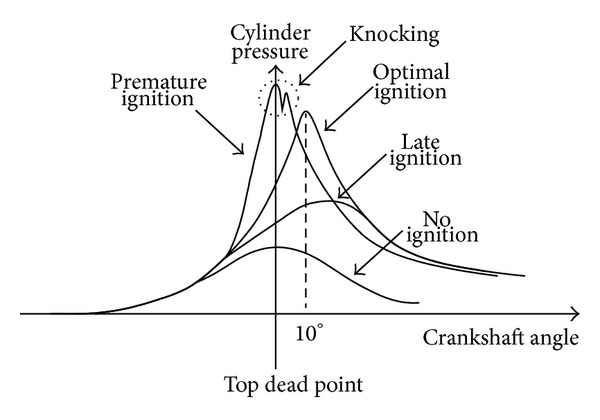
The relationship between cylinder pressure and crankshaft position.

**Figure 4:**
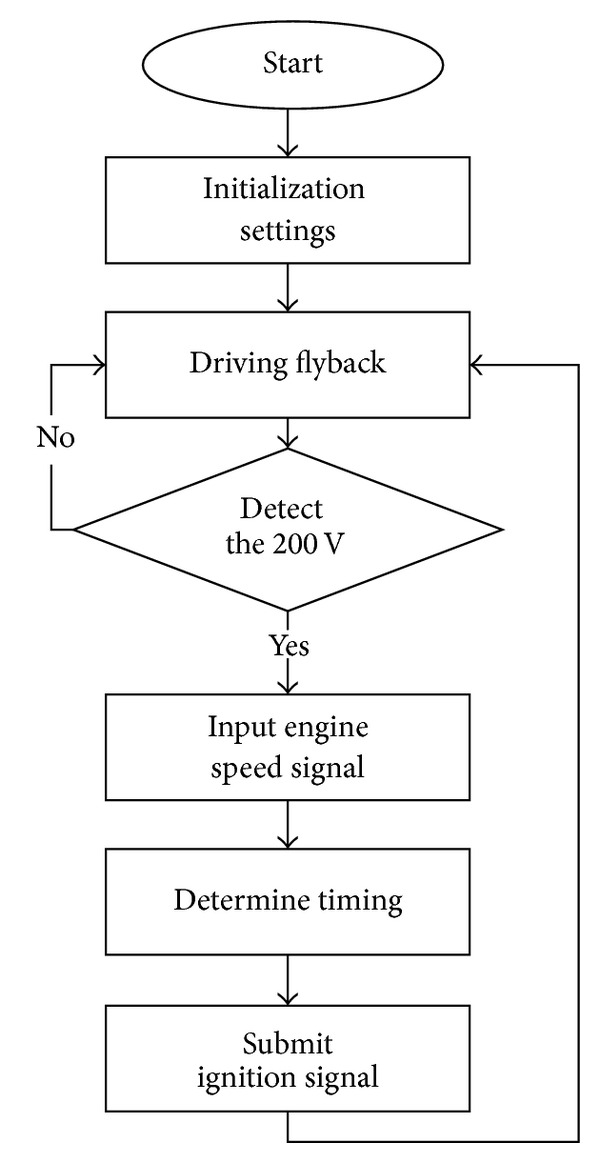
The flowchart of the software programming.

For achieving maximum horsepower output and to avoid knocking, ignition timing has to be controlled exactly. An illustration is shown in Figure 3, which represents the relationship between cylinder pressure and crankshaft position under different igniting conditions. Figure 3 reveals that optimal ignition occurs while engine is ignited at the moment the crankshaft angle is 10 degrees after top dead point. A late ignition or a missing ignition results in lower cylinder pressure, that is, which will lead to more fuel consumption and exhausted gas emission. In Figure 3, even though a premature ignition gains higher cylinder pressure, knocking phenomenon appears. This knocking is in danger of car driving. Therefore, in order to let the engine combustion chamber obtain maximum efficiency, it needs to launch a plug sparkle for the engine after the top dead point of a 10-degree angle. For the optimal 10-degree igniting, a corresponding igniting sequence should be determined instantaneously during various engine speeds. In this paper, by means of software programming on a microprocessor-based controller and with the detection of engine speed, it can be easily achieved. The flowchart of the software programming is shown in Figure 4.

## 3. Operation Principle

**Figure 5:**
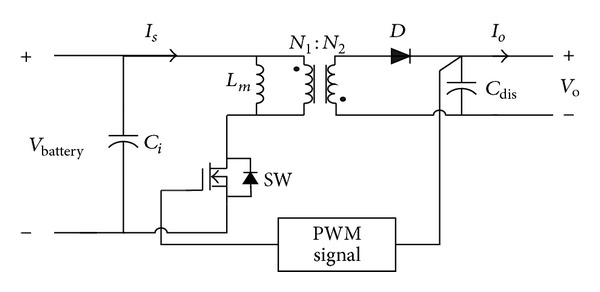
A simplified circuit of the igniter.

The structure of the proposed engine igniter is derived from a flyback converter. By means of PWM controlling and high-frequency switching, the flyback in the igniter delivers battery energy to voltage-stacked capacitor to accumulate energy and voltage in the capacitor. Thus, the main circuit shown in Figure 2 can be simplified as in Figure 5, which is useful to realize the operation of the igniter. The simplified circuit can operate in either CCM (continuous conduction mode) or DCM (discontinuous conduction mode). In this paper, DCM operation is considered.

According to the control for the active switch SW and SCR, the operation principle of the igniter can be divided into seven modes during each ignition cycle, which is described mode by mode in the following.

**Figure 6:**
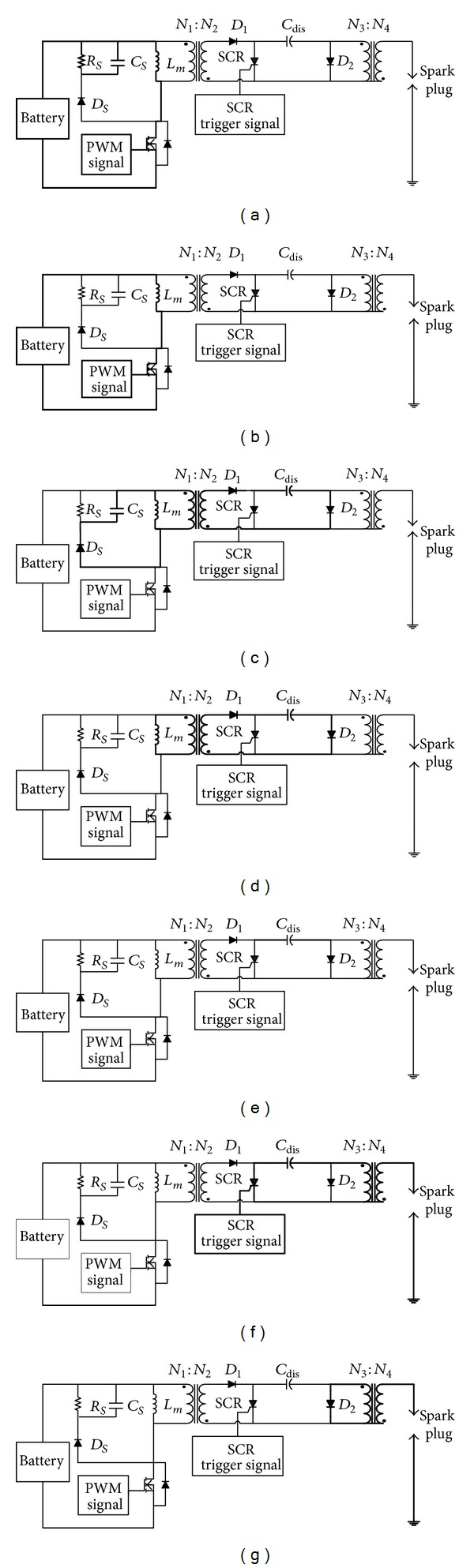
Equivalent circuits for each operation mode over one igniting cycle: (a) mode 1, (b) mode 2, (c) mode 3, (d) mode 4, (e) mode 5, (f) mode 6, and (g) mode 7.

*Mode 1*. As shown in Figure 6(a), the active switch turns on and the battery supplies the magnetizing inductor *L*_*m*_. The current of the inductor increases linearly. Meanwhile, the capacitor *C*_*s*_ in the snubber discharges into the resistor *R*_*s*_.

*Mode 2*. The capacitor *C*_*s*_ discharges energy to the end but the switch SW still stays in on state. The battery continuously stores energy to inductor *L*_*m*_. The equivalent is shown in Figure 6(b).

*Mode 3*. When SW turns off, this mode starts, as shown in Figure 6(c). The voltage across the inductance *L*_*m*_ is reversed. The diodes *D*_1_ and *D*_2_ turn on, and the *C*_dis_ starts to store energy. The leakage inductance energy of the high-frequency transformer is released to the *C*_*s*_ by way of *D*_*s*_. As the current following through leakage inductance drops to zero, this mode ends. 

*Mode 4*. Although the leakage inductance energy is released completely, the magnetizing inductance *L*_*m*_ continues to charge capacitor *C*_dis_. This mode is illustrated in Figure 6(d). The energy stored in the capacitor *C*_dis_ is accumulated successively by a sequence of PWM signals to control the active switch SW. That is, the mode 1 to mode 4 will repeat until the voltage across *C*_dis_ approaches 200V enough to ignite. While the 200 V is reached, operation of the igniter enters into the next mode.

*Mode 5*. As shown in Figure 6(e), the capacitor *C*_dis_ is standing by for igniting. This mode ends as the SCR is trigged.

*Mode 6*. After the microprocessor controller receives speed signal, the controller determines optimal firing timing for the SCR. Then, the SCR is closed and the voltage across capacitor *C*_dis_ is boosted by the ignition coil to a much higher voltage. At this time, the spark plug is ignited to flashover. The equivalent circuit is presented in Figure 6(f).

*Mode 7*. The energy stored in the leakage inductance and the magnetizing inductance of the ignition transformer is lasting to release, as shown in Figure 6(g). When SW starts conducting again at the end of mode 7, the operation of the igniter over an igniting cycle is completed.

In design consideration, assume that the turns-ratio *n* of the transformer in the flyback converter is *N*_1_/*N*_2_, the switching period of the SW is *T*_*s*_, and duty ratio of PWM is *D*. The inductance for boundary conduction operation, *L*_*mB*_, can be determined by

(1) $$\begin{array}{c} L_{mB}=\frac{n^{2}\cdot V_{o}}{2\cdot I_{o}}\cdot {\left(1-D\right)}^{2}\cdot T_{s}, \end{array}$$

where *V*_*o*_ is the output voltage and *I*_*o*_ represents the average output current.

If the flyback converter is operated in DCM, the value of the magnetizing inductance *L*_*m*_ must be less than *L*_*mB*_. The input average current *I*_*s*_ is thus calculated as

(2) $$\begin{array}{c} I_{s}=\frac{1}{2}\frac{V_{\text{battery}}}{L_{m}}D^{2}T_{s}, \end{array}$$

in which *V*_battery_ stands for the input dc voltage. The average input power can be found by

(3) $$\begin{array}{c} P_{s}=V_{\text{battery}}I_{s}=\frac{1}{2}\frac{V_{\text{battery}}^{2}}{L_{m}}D^{2}T_{s}. \end{array}$$

That is,

(4) $$\begin{array}{c} L_{m}=\frac{1}{2}\frac{V_{\text{battery}}^{2}\cdot \eta }{P_{o}}D^{2}T_{s}, \end{array}$$

where *η* expresses the efficiency of the flyback and *P*_*o*_ denotes its output power.

## 4. Simulated and Experimental Results

To verify the feasibility and the functionality of the proposed electronic ignition system, a prototype is constructed and then simulations and practical measurements are carried out.

**Figure 7:**
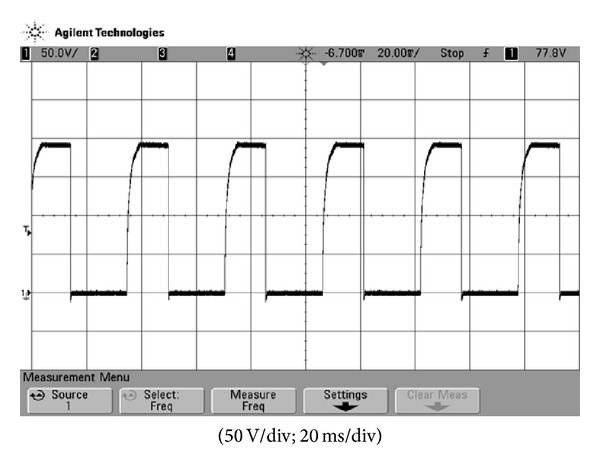
Experimental result of the voltage waveform across capacitor *C*_dis_.

**Figure 8:**
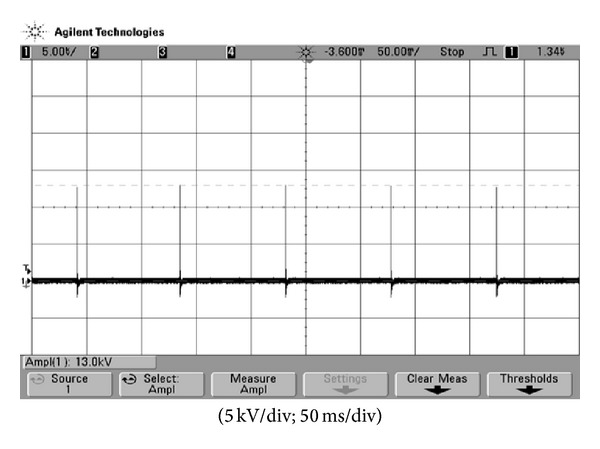
The measured voltage waveform supplying to spark plug.

**Figure 9:**
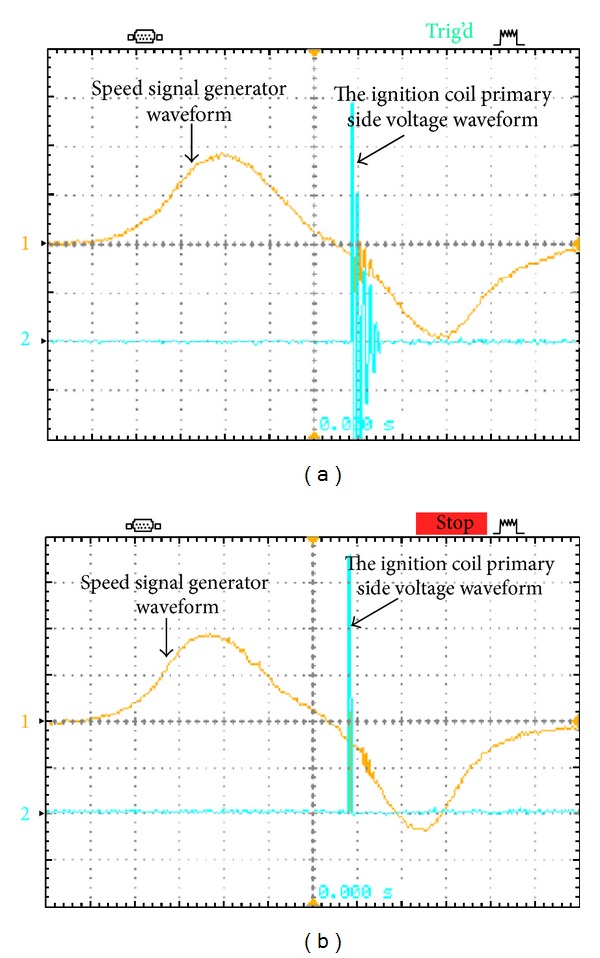
The physical voltage waveforms measured from speed signal generator and ignition coil primary at 1600 rpm: (a) traditional igniter and (b) the proposed igniter.

**Figure 10:**
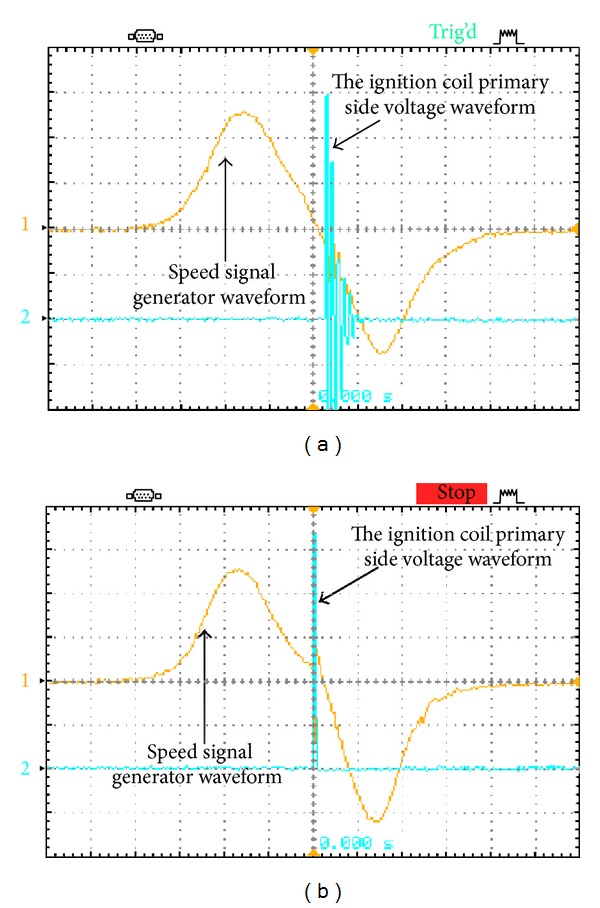
The physical voltage waveforms measured from speed signal generator and ignition coil primary at 2200 rpm: (a) traditional igniter and (b) the proposed igniter.

In the prototype, the battery voltage is 48 V for hybrid electric vehicles and a stacked voltage across *C*_dis_ for igniting is designed as 200 V.  Figure 7 shows the measured voltage waveform of the voltage-stacked capacitor, from which it can be found that before igniting, 200 V can be reached by the flyback. In addition, the rising time of the voltage is just only 5 ms.  Figure 8 is the practical measurement of the voltage supplying to spark plug, from which it can be observed that igniting frequency is stable under fixed speed.  Figure 9(a) shows the voltage waveforms measured from speed signal generator and ignition coil primary of traditional igniter at 1600 rpm, while Figure 9(b) is measured from the proposed igniter.  Figure 9 reveals that at 1600 rpm, even though the traditional igniter meets igniting timing, the following oscillations will degrade combustion efficiency. At 2200 rpm, related measurements are shown in Figure 10. It can be seen that in Figure 10(a) a faster igniting timing cannot be achieved by the traditional one and following oscillation still occurs. On the contrary, in Figure 10(b), the proposed electric igniter not only accomplishes faster timing to complete optimal igniting but also has no oscillation. To demonstrate that the proposed igniter can lead to the reduction of exhaust emission and saving fuel consumption significantly, real-car test is accomplished.  Table 1 is the exhaust emission comparison between the use of traditional igniter and the proposed igniter at 1500 rpm, which are measured by an electric gas analyzer. Meanwhile, physical fuel consumption comparison is shown in Table 2. From Table 1, it can be found that with the use of the proposed igniter the exhaust emissions of HC and CO can be reduced significantly. Table 2 demonstrates that average fuel consumption is saved by 9.252%.

## 5. Conclusions

In this paper an electric igniter derived from flyback converter is proposed, of which igniting timing is programmed by a microprocessor-based controller. According to the speed of vehicle, the controller can determine an optimal timing to ignite so as to improve combustions efficiency, reduce fuel consumption and lower exhaust gas pollution. The structure of the electric igniter is simple and can be powered by the vehicle battery directly. Thus, it is cost-effective and easy to install. In addition, unlike traditional igniter, the proposed igniter has no electric contact so that it can overcome the demerits such as electrode wearing, plug aging, and wrong igniting timing. In this paper, practical measurements and real-car test have verified that the proposed igniter gains higher stability for engine moving, lowers fuel consumption, and reduces exhaust gas emission effectively. That is, it is an environmentally friendly product.

## Figures and Tables

**Table 1:** Physical exhaust emission comparison between the installations of traditional igniter and proposed igniter.

The comparison of exhaust HC and CO for a 125 CC motor at 1500 rpm				
Using traditional igniter		Using the proposed igniter		Comparison results
The 1st measurement of exhaust gas		The 1st measurement of exhaust gas		HC: $\frac{187-108}{187}\times 100\%=42.25\%$ reduced CO: $\frac{1.61-1.30}{1.61}\times 100\%=19.25\%$ reduced
HC (ppm)	181	HC (ppm)	151	
CO (%)	1.69	CO (%)	1.52	

The 2nd measurement of exhaust gas		The 2nd measurement of exhaust gas		HC: $\frac{181-151}{181}\times 100\%=16.57\%$ reduced CO: $\frac{1.69-1.52}{1.69}\times 100\%=10.06\%$ reduced
HC (ppm)	181	HC (ppm)	151	
CO (%)	1.69	CO (%)	1.52	

The 3rd measurement of exhaust gas		The 3rd measurement of exhaust gas		HC: $\frac{196-148}{196}\times 100\%=24.49\%$ reduced CO: $\frac{1.83-1.55}{1.83}\times 100\%=15.30\%$ reduced
HC (ppm)	196	HC (ppm)	148	
CO (%)	1.83	CO (%)	1.55	

**Table 2:** Physical fuel consumption comparison between the installations of traditional igniter and proposed igniter.

The physical measurements of fuel consumption from a 125 CC motor			
	Mileage (km)	Fuel consumption (liter)	Calculation
With traditional igniter			
1	1708–1820	4.43	Total trip: 386 km Fuel consumption: 14.88 liters Average: 25.94 km/liter
2	1820–1923	3.53	
3	1923–2001	3.53	
4	2001–2094	3.39	

With the proposed igniter			
1	2670–2736	3.27	Total trip: 280 km Fuel consumption: 9.88 liters Average: 28.34 km/liter
2	2736–2861	3.27	
3	2861–2882	1.67	
4	2882–2950	1.67	

The percentage of fuel saving is 9.252%.			$\frac{28.34-25.94}{25.94}\times 100\%=9.252\%$ reduced
